# Tobacco Dependence Treatment Grants: A Collaborative Approach to the Implementation of WHO Tobacco Control Initiatives

**DOI:** 10.1155/2018/8429738

**Published:** 2018-03-22

**Authors:** Margaret B. Nolan, Katherine E. Kemper, Thomas J. Glynn, Richard D. Hurt, J. Taylor Hays

**Affiliations:** ^1^Department of Anesthesia, Nicotine Dependence Center, Mayo Clinic, Rochester, MN, USA; ^2^Nicotine Dependence Center, Mayo Clinic, Rochester, MN, USA; ^3^Department of Medicine, Stanford Prevention Research Center, Stanford University, Stanford, CA, USA; ^4^Department of General Internal Medicine, Nicotine Dependence Center, Mayo Clinic, Rochester, MN, USA

## Abstract

The number of global tobacco-related deaths is projected to increase from about 6 million to 8 million annually by 2030, with more than 80% of these occurring in low- and middle-income countries (LMICs). The World Health Organization Framework Convention on Tobacco Control (FCTC) came into force in 2005 and Article 14 relates specifically to the treatment of tobacco dependence. However, LMICs, in particular, face several barriers to implementing tobacco dependence treatment. This paper is a descriptive evaluation of a novel grant funding mechanism that was initiated in 2014 to address these barriers.* Global Bridges*. Healthcare Alliance for Tobacco Dependence Treatment aims to create and mobilize a global network of healthcare professionals and organizations dedicated to advancing evidence-based tobacco dependence treatment and advocating for effective tobacco control policy. A 2014 request for proposals (RFP) focused on these goals, particularly in LMICs, where funding for this work had been previously unavailable. 19 grants were awarded by Global Bridges to organizations in low- and middle-income countries across all six WHO regions. Virtually all focused on developing a tobacco dependence treatment curriculum for healthcare providers, while also influencing the political environment for Article 14 implementation. As a direct result of these projects, close to 9,000 healthcare providers have been trained in tobacco dependence treatment and an estimated 150,000 patients have been offered treatment. Because most of these projects are designed with a “train-the-trainer” component, two years of grant funding has been a tremendous catalyst for accelerating change in tobacco dependence treatment practices throughout the world. In order to foster such exponential growth and continue to maintain the impact of these projects, ongoing financial, educational, and professional commitments are required.

## 1. Introduction

According to the 2016 World Health Organization/National Cancer Institute Monograph, a global report on tobacco control, the number of global tobacco-related deaths is projected to increase from about 6 million to 8 million annually by 2030, with more than 80% of these occurring in low- and middle-income countries (LMICs) [1]. These are more annual deaths worldwide than are caused by HIV/AIDS, TB, and malaria combined [2]. Moreover, the economic burden of tobacco use is staggering, with the total cost of smoking (including productivity losses from death and disability) reaching more than $1.4 trillion US dollars per year—equivalent to 1.8% of the world's annual GDP [3].

Though prevalence of smoking in the US is currently around 15% of the adult population, prevalence rates in many other countries, particularly LMICs, can reach over 40%. These high prevalence rates pose a challenge for countries to create a policy climate conducive to tobacco control measures, particularly when it comes to developing the infrastructure necessary to build and maintain a national tobacco dependence treatment program. Evidence from a recent survey suggests that there are additional barriers to implementing tobacco dependence treatment, particularly in LMICs, including perceived costs of providing tobacco dependence treatment support and a lack of clarity about the effectiveness and cost effectiveness of tobacco dependence treatment [4].

In 2005, the world's first public health treaty, the World Health Organization Framework Convention on Tobacco Control (FCTC), came into force and has since become one of the most widely embraced treaties in United Nations history; 180 Parties have now ratified the treaty and are making progress to address the global tobacco epidemic. FCTC Article 14 relates specifically to the treatment of tobacco dependence and encourages countries to

1. strengthen or create a sustainable infrastructure which motivates attempts to quit, ensure wide access to support for tobacco users who wish to quit, and provide sustainable resources to ensure that such support is available;
2. identify the key, effective measures needed to promote tobacco cessation and incorporate tobacco dependence treatment into national tobacco control programs and healthcare systems;
3. share experiences and collaborate in order to facilitate the development or strengthening of support for tobacco cessation and tobacco dependence treatment [5].

As of the 2016 Global Progress Reports by the WHO, only 50% of countries have even* begun* implementation of Article 14 [6]. More detailed surveys of participating Parties undertaken by Raw and colleagues reveal that LMICs had significantly less tobacco dependence treatment provision than high-income countries as of 2012 [4] There has been little progress in this area as of 2017, with only one low-income country reporting a national quitline and nicotine replacement therapy or cessation services that are partially cost-covered [7]. Low-income countries that had ratified the treaty also had low rates of national guideline development as of 2012 (11% versus 75% in high-income countries of ratification), suggesting that guidelines for tobacco dependence treatment were not being prioritized in the health policy of LMICs [4].

*Global Bridges*. Healthcare Alliance for Tobacco Dependence Treatment was created in 2010 with an Investigator Initiated Education Grant to Mayo Clinic from Pfizer Independent Grants for Learning and Change (IGLC). The primary mission of Global Bridges is to create and mobilize a global network of healthcare professionals and organizations dedicated to advancing evidence-based tobacco dependence treatment and advocating for effective tobacco control policy. In considering whether to enter into a partnership with a commercial entity, the Global Bridges leadership weighed the potential benefit of addressing the tremendous global health burden of tobacco and lack of other funding sources against the theoretical cost of being identified as working with an industry sponsor. To minimize potential disadvantages, a firewall was created to limit the funder's involvement in decisions regarding grant applications and awards. All grant awards were reviewed and scored by an independent scientific review panel responsible for all final funding approvals. The corporate sponsor had one representative on the 8-member scientific review panel while the remaining members represented Global Bridges and Mayo Clinic (5) along with 2 external experts.

 As the goals of Global Bridges and FCTC Article 14 are closely aligned, the opportunity arose for Global Bridges grant funding to aid participating countries in the implementation of FCTC Article 14. Global Bridges aims to help such countries not only to train healthcare professionals to deliver tobacco dependence treatment interventions, but also to create and foster a policy environment conducive to the development and fulfillment of national tobacco dependence treatment efforts aligned with Article 14 mandates. For this reason, a request for proposals (RFP) from Global Bridges was released in 2014 and focused on three project categories: (1) expansion of preexisting capacity building programs with demonstrated success; (2) creation of healthcare professional advocacy programs for Article 14 implementation; and (3) development of new healthcare professional training programs, particularly in LMICs, in which funding for this work had been previously unavailable. This paper is a descriptive evaluation of the projects resulting from this round of 2-year grants.

In response to the 2014 RFP, a total of 19 projects were funded which vary considerably in scope and design, but all focusing on the three primary themes outlined in the RFP, each aimed at developing support for national tobacco dependence treatment strategies. This funding opportunity is novel, since it is the first international funding program that has emphasized the role of* treatment* of tobacco dependence as an integral part of comprehensive tobacco control efforts.

Not only does treatment result in more successful quitting and therefore years of life saved for individual patients, but it also begins to lessen the cost burden of noncommunicable diseases (NCDs) in countries least able to afford them. As Raw and colleagues explain, preventing people from starting to smoke will save lives years down the line, but “…only effective tobacco cessation will have a sufficient effect on mortality in the few years left to reach the UN/WHO goal of a 25% reduction in premature mortality from NCDs by 2025” [8]. Compared to other preventive health interventions, smoking cessation treatment is extremely cost-effective, even in higher income countries where healthcare costs are greater. The cost of a brief tobacco intervention per healthy life-year gained for low-income countries, for example, is less than $100 per year, and for high-income countries, only about $4,200 per life-year gained [9]. To put this in context, the historic threshold for a cost-effective health intervention in the United States is $50,000 per quality-adjusted life-year gained [10].

**Figure 1:**
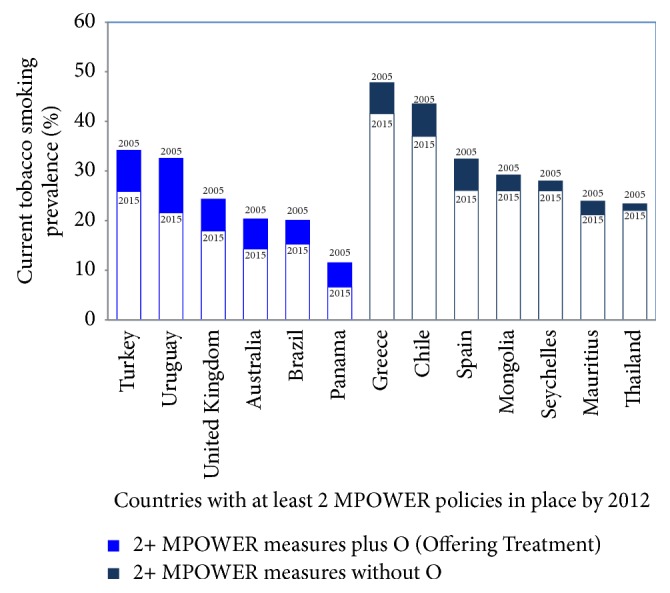
10-year change in smoking prevalence in countries with strong cessation programs versus those who have focused on other MPOWER elements of tobacco control. Source: data compiled from WHO Report on the Global Tobacco Epidemic, 2013, and the WHO Report on Trends in Tobacco Smoking, 2000–2025. Unpublished, by personal communication with Dongbo Fu. World Health Organization, Geneva, Switzerland, 2012.

In addition to being cost-effective, tobacco dependence treatment can also have a synergistic effect with other preventive measures, such as clean indoor air policies or cigarette taxation [7]. Countries that have included tobacco treatment services in their tobacco control plans (the “O” in the WHO acronym MPOWER—Monitor, Protect, Offer (treatment), Warn, Enforce, and Raise (taxes)) have been able to reduce smoking prevalence over a relatively short period of time compared to those who have focused on other elements of tobacco control (Figure 1). Unfortunately, population-level measures have instead been emphasized at the expense of creating national infrastructure to support tobacco dependence treatment and training healthcare personnel to treat patients who are already addicted to tobacco. A 2013 survey assessing the progress of WHO Article 14 implementation in 84 countries found that 21% (1 in 5) still had no program in place to train individuals to provide tobacco dependence treatment [11]. The WHO Report on the Global Tobacco Epidemic, 2017: Monitoring Tobacco Use and Prevention Policies reports that 67% of the world's population still has no access to tobacco cessation services [7]. Although the number of countries reporting at least one MPOWER measure implemented at the highest level of achievement has increased from 107 to 121 (increasing coverage from 2.9 to 4.7 billion people from 2007 to 2016), the increase in tobacco cessation services provision has been relatively small. Only about 12%, or 26 countries, report having complete smoking cessation services in place, whereas nearly 30% (55 countries) report complete policies for clean indoor air and 40% (78 countries) for warning labels [7]. If countries continue to implement these policies that encourage people to* want* to quit smoking, then it becomes a moral imperative to simultaneously improve access to tobacco cessation treatment services.

Tobacco dependence treatment, therefore, while not often the focus of tobacco control funding, could have the potential to quickly and dramatically change the chronic disease landscape in LMICs.

## 2. Materials and Methods

In 2014, nineteen grants were awarded by Global Bridges to organizations in low- and middle-income countries across all six WHO regions (Table 1), with goals for Article 14 implementation between 2014 and 2016. Many of the grant projects use the “train-the-trainer” approach to capacity building for tobacco dependence treatment, creating a pool of qualified instructors who are able to disperse the evidence-based principles underpinning effective tobacco dependence treatment to healthcare teams within a country or a geographic region. Many of the training programs also include innovative solutions to address the barriers of tobacco dependence treatment in many LMICs, such as a distance learning component that allows healthcare providers or community health workers from rural or underserved areas to fully participate in training modules. Others focus on training healthcare providers who care for specific underserved populations, such as patients with severe mental illness or residents of remote geographical areas. The effects of this work are constantly evolving and expanding as more and more healthcare providers are trained in tobacco dependence treatment, practice it in their daily work, and share their knowledge with others.

### 2.1. The 2.1. Target Populations

All grants target healthcare providers across a broad spectrum from physicians and dentists to community health workers, either directly through training programs, or indirectly via policymakers. Due to the diversity of language, culture, and existing infrastructure for tobacco dependence treatment in each country, the types of healthcare professionals targeted have varied. Most projects have targeted primary care physicians as the largest segment of the physician workforce, including medical school trainees and primary care residents, in order to introduce tobacco dependence treatment as a routine part of general medical practice. These providers then became leaders of tobacco treatment practice and served as a model for their peers and colleagues at other institutions. Projects that targeted medical universities were able to then incorporate this training into their standard curriculum.

Other healthcare providers targeted in these programs include dentists, nurses, psychologists, pharmacists, and community health workers (see Table 1 for a list of projects by target population). By training this diverse body of health professionals and allied community workers, the capacity of a country or region to treat tobacco dependence is greatly increased and provides for exploring approaches that best meet local needs (e.g., in many countries community health workers are much more accessible than are licensed health professionals). A few projects focused on the policymakers who were responsible for creating a political environment conducive to the implementation of FCTC Article 14—a necessary starting point for some countries with nascent tobacco control infrastructure.

A variety of techniques and technologies were used to conduct healthcare provider training sessions and disseminate knowledge related to tobacco dependence treatment. Training sessions were in-person, online, or a combination of in-person with a virtual component for continued support and reference, such as an online resource or text messaging support system. Several projects used training packages designed by the WHO on “Building Capacity for Tobacco Control,” or competencies from the Association for the Treatment of Tobacco Use and Dependence (ATTUD) [12], adapting them to fit their country's language and healthcare model. Most projects that used the tailored curriculum delivered it with in-person training sessions from 1 to 3 days in length, which either focused on the “train-the-trainer” approach, creating a cohort of qualified trainers to disseminate their knowledge to other primary care providers, or focused on training groups of healthcare professionals who would be directly providing treatment to their patients. Some projects did both, such as that of the InterAmerican Heart Foundation in the Latin American Region and the National Heart Foundation in Bangladesh, in order to maximize the impact of their training sessions. All training sessions included an evaluation component, usually through a pretest/posttest mechanism that most commonly measured attitudes, knowledge, skills, and clinical practice behavior changes they intended to make. Due to limits on both time and resources, these projects were necessarily limited in scope. Long term data on patient outcomes and policy achievements could not be collected within the project period, though one project applied for a funding extension and others have been able to secure funding from other sources, which may increase the potential to measure longer-term impact.

The technology used in these projects varied according to practical considerations such as its availability in each country. In Nigeria, text messaging was used to reinforce initial messages from physician training sessions and to recruit a secondary group of physicians to begin addressing tobacco use in their practice via low-intensity messaging reminders. Online training curricula were used in several projects, particularly those that required administering curriculum in multiple languages, or in settings where in-person training sessions were limited due to geographic or financial constraints. Such technology, however, required working computers or mobile devices and reliable Internet connections, which are not always available in low-resource settings. Finally, the reach of tobacco treatment interventions was extended through the use of mass media in some countries, such as Uganda, where radio talk shows and advertisements were a key component of spreading the word about available tobacco treatment services.

### 2.2. Estimating Project Impact

Grantees were asked to submit periodic project updates and a final project report at the completion of the 2-year funding cycle. Global Bridges, as the coordinating center, reviewed each final report and abstracted relevant outcomes data. Program directors were contacted for clarification of methods or results, if needed, but due to a lack of a standard reporting system for outcomes data and a diverse array of project aims, the outcomes data was incomplete. Instead of combining data collected by different methods for different purposes, the evaluation of the grant program itself was performed by considering several outcomes, such as the reach of provider training programs, government interest and involvement in Article 14 implementation, and dissemination of knowledge via peer-reviewed publications, posters, presentations, or mass media campaigns.

## 3. Results

Seed money in the setting of LMICs, where many grantees started with very little existing tobacco treatment infrastructure, necessarily is limited by time constraints and logistical hurdles. Despite this, 56 of the 61 grantee specific aims proposed prior to funding allocation were successfully achieved within the grant period, and 5 are in progress or near completion. The main measurable grant outcomes are summarized in Table 2.

Projects that involved training programs for healthcare professionals (18/19 grants) reported the number of healthcare providers impacted, as well as outcome measures related to the training sessions themselves. The most commonly measured outcomes included (1) provider knowledge about tobacco treatment services (including the benefit of brief provider advice and pharmacotherapy) in a pretest/posttest comparison, (2) extent of practice of asking, advising, and referring patients, (3) intent to use these practices in the future, and (4) postintervention practice of skills learned. In most cases, an estimate of patients reached was also provided, but the methods for this estimate varied by project. The training sessions were universally effective in showing at least short term improvement in all domains measured (Table 2).

As a direct result of these projects, close to 9,000 healthcare providers have been trained in tobacco dependence treatment and over 150,000 patients are estimated to have been offered treatment. Many of the training programs started as a result of these projects were designed to be perpetuated and expanded by creating an additional generation of tobacco treatment advocates. Providers who are now qualified trainers are able to train other healthcare professionals to perform similar services. For this reason, the number of providers and patients impacted by these projects continues to grow. For example, as part of the InterAmerican Heart Foundation's work in Latin America, it was estimated that if each healthcare worker trained during this project offered just one patient brief advice each working day, it would result in over 43,000 patients receiving brief advice and over 1,000 successful quitters per year.

The curricula designed to train healthcare providers in these projects are now being incorporated into ongoing training programs in the project countries and elsewhere, many as part of residency and CME programs, further expanding the reach of tobacco dependence treatment. The EuroPean Accreditation Curriculum on Tobacco Treatment (EPACTT) project, for example, developed and expanded such a Global Bridges-supported curriculum throughout Romania, Armenia, Georgia, Ukraine, and Russia via e-courses that can be accessed through any computer or mobile device, allowing physicians to earn Continuing Medical Education (CME) credits and become accredited providers of tobacco treatment services. In Bangladesh, seven dedicated tobacco dependence treatment clinics have been established throughout the country to maximize the reach of these services, along with the initiation of a national quitline, which can help increase that reach further. And in the Eastern Mediterranean region, where the program developed by the King Hussein Cancer Center is one of the only programs outside the US to be awarded accreditation through the Council for Tobacco Treatment Training Programs, functioning hubs with tobacco dependence treatment clinics have been established in Egypt, Tunisia, Oman, and Morocco, supported by strong national institutions (cancer centers, universities) and Ministries of Health.

## 4. Discussion

In virtually all countries, advocacy and raising awareness of the need for tobacco treatment interventions are a necessary starting point for implementing FCTC Article 14. For this reason, other important outcomes were measures of public awareness, media coverage, publications, and presence at government meetings and discussions with Ministries of Health. Some projects were able to work closely with the national Ministry of Health to design a curriculum for tobacco dependence treatment that could be adopted on a national level, while others focused on a needs-assessment and advocacy platform for inspiring policymakers to prioritize Article 14 implementation.

Though these types of projects present measurement challenges, they can have significant future impacts on tobacco dependence treatment capacity. Some examples of projects with high potential impact and plans for sustainability include the following:

1. In China, the Center for Tobacco Control Research reported that news of a workshop training tobacco treatment champions from universities throughout the country was released by the “State Council Information Office of the People's Republic of China” to 21 news agencies and then forwarded to more than 200 news agencies and websites throughout the nation. Such widespread attention to, and implied official endorsement of, tobacco dependence treatment can be a powerful force impacting the political environment for implementing changes in clinical practice and conveying a message to the public that addressing tobacco use is a priority.
2. In Greece, though initial goals were to train primary care providers to perform tobacco interventions, the high prevalence of smoking among the physicians themselves became a barrier to their training and presented an opportunity for intervention. The “TiTAN Crete” project was able to support smoking abstinence among general practitioners who were tobacco users by delivering a group counseling session, subsequently reporting that 75% of the providers who reported tobacco use at baseline were abstinent at follow-up.
3. In a training project aligned with China's smoke-free workplace initiative, conducted by the China Center for Disease Control and the ThinkTank Research Center, the grantee initially expected 30 companies in each of three cities in China to participate in their workshops. Instead, over 140 workplaces attended the workshops. Forty of these companies had over 1,000 employees each and all companies together included over 100,000 employees. At their follow-up, 87% of enterprises reported that their smoking rate had been reduced and at least 1550 employees reported that they had stopped smoking.
4. The King Hussein Cancer Center (KHCC) conducted a project to expand availability of tobacco dependence treatment services in five countries in the Eastern Mediterranean Region: Jordan, Egypt, Tunisia, Morocco, and Oman. KHCC identified leadership “host” organizations in each country and developed evidence-based, culturally appropriate curricula in three languages. A national treatment guideline was also developed in cooperation with the Jordanian Ministry of Health and WHO. To date, 76 trainers have been equipped to deliver training to HCPs in their country; through an extension to the project, training sessions were being held throughout 2017.
5. The small (population 10.7 million; tobacco users 2.2 million) Latin American country of Bolivia began with virtually no tobacco control infrastructure. Accordingly, the focus of this policy grant was to conduct a National Situation Analysis with the Health Ministry and international tobacco control experts and adopt a tobacco control strategy including a cessation plan and monitoring mechanism. At the request of the Health Ministry, the proposed plan was expanded to include other tobacco control measures. Also and separately, as a result of the initial work on this project, additional funders provided support for infrastructure (a dedicated office of the regional grantee organization was established in Bolivia) and smoke-free policy work.

## 5. Conclusions

Because most of these projects are designed with a “train-the-trainer” component, two years of grant funding can be a tremendous catalyst for accelerating change in tobacco dependence treatment practices throughout the world. In order to foster such exponential growth and continue to maintain the impact of these projects, ongoing financial, educational, and professional commitments are required.

### 5.1. Lessons Learned

Despite the many strengths of this grant program, there are ways in which it can be improved going forward that may serve as a model to other similar tobacco control programs. For example, a standard outcomes reporting requirement for grantees, designed and distributed prior to grant funding being provided, will be essential going forward. This will allow for more standard planning of outcomes measures on the part of grantees and stronger evaluation of project progress and impact on the part of Global Bridges, as the coordinating center. The amount and duration of funding available, however, must be recognized as “seed money” to start building a framework for tobacco treatment services, since 2 years is not enough time to build the infrastructure necessary to realize and measure longer-term outcomes like practice change and population abstinence outcomes. Recognizing the goals and aims of these grants are novel, but limited in scope, is also an essential part of the program's success.

### 5.2. Future Directions

Anticipating the need for continued self-sufficiency, many projects feature built-in mechanisms for sustainability, such as the continued updating of online training modules to incorporate current evidence-based treatment guidelines. Others that focus more on the policy environment have worked to put tobacco dependence treatment “on the radar” for Ministries of Health and other governing and policy-making bodies who prioritize funding and resource management. Their sustainability therefore relies upon continued advocacy in policy settings. And several projects have been extended to include a second phase, with funding from the original source and/or different funders, where lessons learned from the first training project are being incorporated for continued expansion of treatment services. Subsequent Pfizer-funded programs have been announced, including eleven funded projects in Europe which are beginning in 2017, and a request for proposals for projects in Japan. The international network of experts whose foundation was laid with this project continues to expand and facilitate the sharing of learning, to maintain these advances and spread the knowledge and skills of tobacco dependence treatment throughout the world.

## Figures and Tables

**Table 1:** Summary of Global Bridges grantees, 2014–2016.

Organization name	Project title	Country	WHO region	Target population
International Primary Care Respiratory Group	Training Community Health Workers in Rural Uganda to Introduce Stop Smoking Interventions in the Context of a Lung Health Awareness Campaign	Uganda	Africa	General practitioners, physician assistants, and community health workers

University of Nairobi	Tobacco Cessation through Use of Oral Health Care Providers in Kenya	Kenya	Africa	Oral health care providers and policy makers

College of Medicine, University of Lagos	Physicians as Change Agents to Facilitate Tobacco Cessation in Clinical Practice	Nigeria	Africa	Resident physicians and dentists

InterAmerican Heart Foundation	Capacity Building for Smoking Cessation Training in Latin America: Expanding the Work of Global Bridges 2011–2013	Latin America Region	Americas	Physicians and allied health staff

InterAmerican Heart Foundation	Strengthening Healthcare Capacity for Article 14 by Developing a Strategic Approach to Analyzing Need and Planning a Strategy	Bolivia	Americas	Policymakers, primary care physicians, nurses, dentists, nutritionists, and psychiatrists

Fundación Interamericana del Corazón México	Strengthening Healthcare Capacity for FCTC Article 14 Implementation in Mexico by Advocating for a More Strategic Approach to Expanding Tobacco Dependence Treatment	Mexico	Americas	Policymakers

Catalan Institute of Oncology	Development and Dissemination of a Tobacco Cessation Training Program for Healthcare Professionals in Spanish-Speaking Countries	Guatemala, Paraguay, Bolivia	Americas	Healthcare providers

Centro de Estudos em Saúde Mental do ABC	Implementing Evidence-Based Tobacco Dependency Treatment in Addiction/Mental Healthcare Units in Brazil	Brazil	Americas	Physicians, psychologists, nurses, social workers, pharmacists, and dentists

European Network for Smoking and Tobacco Prevention (ENSP)	EPACTT-EuroPean Accreditation Curriculum on Tobacco Treatment	Romania, Armenia, Georgia, Ukraine, Russia	Europe	Healthcare providers and policymakers

University of Crete	Developing a Primary Care Tobacco Dependence Treatment Network in Crete, Greece	Greece	Europe	Primary care providers

University of Arizona	Building Capacity for Illness-Specific Tobacco Cessation among Nurses and Clinical Psychologists in Turkey	Turkey	Europe	Nurses and clinical psychologists

American University of Armenia, School of Public Health	Implementing the FCTC Article 14 in Armenia through Advocacy and Training	Armenia	Europe	Family physicians and general practitioners, policymakers

National Heart Foundation Hospital & Research Institute	Capacity Building of Primary Care Physicians for Treatment of Tobacco Dependence in Bangladesh	Bangladesh	SE Asia	Primary care physicians, nurses, and community health workers

Public Health Foundation of India	Strengthening Cessation Capacity in the National Tobacco Control Programme of India	India	SE Asia	Physicians (primary and secondary healthcare facilities)

Salaam Bombay Foundation	Capacity Building of Healthcare Professionals to Create a Workforce Trained in Tobacco Dependence Treatment at Different Levels of Healthcare Settings	India	SE Asia	Healthcare providers (medical and dental)

Zhejiang University	Building Tobacco Treatment Capacity in Medical Universities and Affiliated Hospitals in China	China	Western Pacific	Nurses, general practitioners, and other physicians

China–United States Smoke-free Workplace Initiative	Build the Bridges: From Capacity Building to Practice	China	Western Pacific	Healthcare providers (physicians) and corporations

Institute of Social and Medical Studies	Building Capacity to Deliver Evidence Based Tobacco Use Treatment in Vietnam	Vietnam	Western Pacific	Healthcare providers and Policymakers

King Hussein Cancer Center	Expand Availability of Tobacco Dependence Treatment Services in the Eastern Mediterranean Region through Building Sustainable Evidence-Based in-Country Training Programs	Jordan, Oman, Tunisia, Morocco, Egypt	Eastern Mediterranean	Healthcare providers

**Table 2:** Outcomes of the 19 grants and their reported data in 2016.

Outcomes	Number of grants (*n* = 19)	Measured outcome
Program impact outcomes		
(i) Trained healthcare providers	18	8,854 HCPs trained
(ii) Patient impact estimate	16	157,281 patients counseled^*∗*^
Curriculum characteristics		
(i) Curriculum design focus	17	190 hours of designed curriculum
(ii) Train-the-trainer component	9	1,435 master trainers trained
(iii) Distance learning component	9	46 online curriculum hours designed
Learner outcomes		
(i) Assessed knowledge acquisition	14	14/14 significant improvement before/after training
(ii) Assessed clinical practice change	13	13/13 significant practice change before/after training
Dissemination outcomes		
(i) Mass media campaigns	2	
(ii) Peer-reviewed publications	12	11 peer-reviewed manuscripts published, 15 in progress
(iii) Oral or poster presentations	19	67 presentations
Political outcomes		
(i) Collaboration with MOH	7	3 developed National Guidelines

^*∗*^Methods of patient reach estimates varied by project, and most were not measured directly.
